# Locally Delivered Umbilical Cord Mesenchymal Stromal Cells Reduce Chronic Inflammation in Long-Term Nonhealing Wounds: A Randomized Study

**DOI:** 10.1155/2020/5308609

**Published:** 2020-02-14

**Authors:** Yulia Suzdaltseva, Sergey Zhidkih, Sergey L. Kiselev, Victor Stupin

**Affiliations:** ^1^Vavilov Institute of General Genetics, Russian Academy of Sciences, Moscow, Russia; ^2^Department of Hospital Surgery, Pirogov Russian National Research Medical University, Moscow, Russia; ^3^Department of Purulent Surgery, Municipal Clinical Hospital №15, Moscow, Russia

## Abstract

Inflammation is part of a complex biological response to injury that mediates a rapid mobilization of cells and triggers the restoration of tissue homeostasis. The systemic diseases of the connective tissues, repetitive strain injuries, neuropathy, and vascular impairment lead to the development of a chronic inflammatory state. In such cases, a forced intervention is required to trigger tissue regeneration. Mesenchymal stromal cells (MSCs) have been considered a perspective tool for regenerative medicine because of their ability to change the expression and secretory profile under the influence of signals from the microenvironment to perform a regulatory function at the site of tissue damage. In this study, MSCs were isolated from the human umbilical cord (UCMSCs). The ability of UCMSCs to regulate chronic inflammation was investigated in a randomized placebo-controlled pilot study to assess the efficacy and safety of UCMSC therapy in patients with nonhealing wounds. A total of 108 patients with chronic wounds of different etiologies were randomly divided into two groups according to the criteria of inclusion and exclusion. The group (*n* = 59) that was treated with a single local subcutaneous infusion of UCMSCs around the wound periphery showed a pronounced growth of granulation tissue, improved blood microcirculation, and reduction in wound size compared to the placebo group (*n* = 49). No prominent adverse events were detected in patients from the UCMSC group during the 1-year follow-up period. This research has demonstrated that locally delivered allogeneic UCMSCs can contribute to chronic wound repair and provide an additional support toward new therapeutic strategies. Registration certificate №FS2006/341 was issued by the Federal Service for Surveillance in Healthcare.

## 1. Introduction

**Figure 1:**
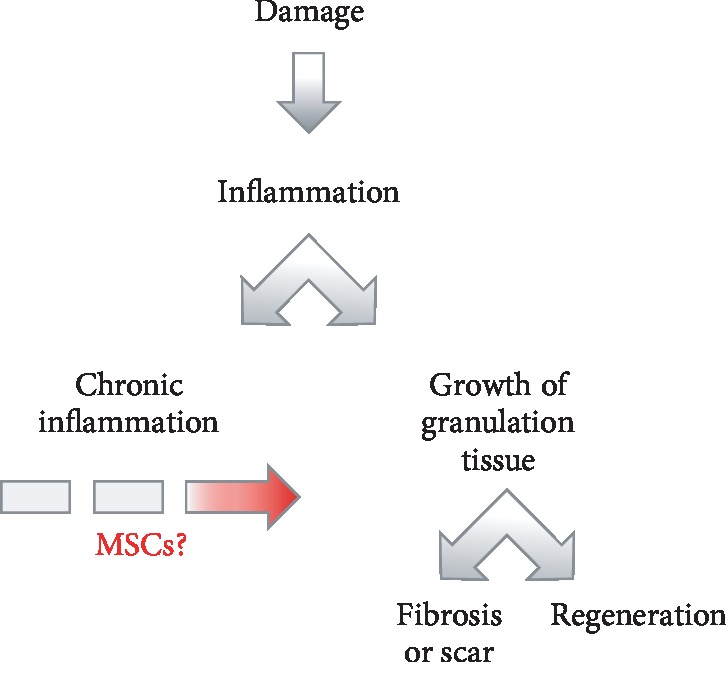
Pathophysiological variants of case scenario in tissue repair after injury. The best case scenario for wound healing is tissue regeneration—the complete repair of structure and function. Adults usually experience wound healing with various degrees of scar formation. Tissue repair does not occur if chronic inflammation develops. The strategy to control the inflammatory response is a shift in the balance from chronic inflammation toward tissue regeneration.

Wound healing is a highly ordered and orchestrated process that includes inflammation, cell proliferation, formation of extracellular matrix, and tissue remodeling [1, 2]. The regeneration of damaged tissue is conditionally subdivided into two successive phases: inflammation and regeneration. The regeneration phase is also conditionally subdivided into two overlapping stages: (A) proliferation (granulation), which is the creation of temporary granulation tissue, which quickly closes and restores the original form of the damaged area, and (B) remodeling, which refers to the replacement of a temporary supportive structure with permanent tissue that has the corresponding morphological and physiological characteristics of the original tissue. Each subsequent phase of tissue repair is functionally linked to the previous one. Disorders in these relationships lead to the arrest of the tissue repair process in the intermediate stages and to the development of chronic inflammation or fibrosis [3]. The successful completion of the remodeling stage means a full regeneration of tissue architecture and function (Figure 1). The regenerative potential of tissues is known to decline with age [4]. In adults, regeneration is usually completed at the remodeling stage, and skin wounds heal with varying degrees of scar formation [5]. However, in some cases, the inflammation does not jugulate but continues in a chronic form [6]. A forced intervention is then required to prevent the development of chronic inflammation and to trigger the subsequent phase of tissue regeneration. Repetitive strain injuries, neuropathy, chronic venous and arterial insufficiency of the lower limbs, systemic diseases of connective tissue, and chronic persistent local infections can lead to the development of chronic wounds. There are many types of nonhealing wounds, including trophic ulcers of different etiologies, bed sores, and diabetic foot [6–8]. Ulcer treatment to obtain wound closure as expeditiously as possible requires wound cleansing, necrotic tissue debridement, amelioration of damaging factors, including infection management with antibiotics and antiseptics, improvement of the regional venous and arterial blood circulation, and medical management of comorbidities. However, chronic wounds do not heal for a long time and often recur after healing even with intensive treatment. Today, the greatest prospects in the treatment of chronic ulcers are associated with the use of cell-based therapies [9–11].

Mesenchymal stromal cells (MSCs) have been considered a perspective tool for regenerative medicine. MSCs have attracted much attention because of their capacity for proliferation and migration and their multilineage differentiation into osteoblasts, chondroblasts, and adipocytes. Recent investigations have indicated that MSCs play a crucial role in regulating almost all stages of inflammation and tissue regeneration; this has been confirmed by experimental data in vitro, studies on animal models, and clinical trials [12]. During inflammation, MSCs exert multiple paracrine and immunomodulatory effects to control the course of this process and its transition to subsequent stages of tissue repair [13–16]. Under the influence of external signals from the microenvironment, MSCs can dynamically change their expression and secretory profiles and, therefore, perform a regulatory function relative to immune cells at the site of inflammation [17]. At the stage of regeneration, MSCs synthesize and remodel the extracellular matrix, stimulating angiogenesis and reepithelialization [18, 19]. However, the spatiotemporal dynamics of these effects, as well as the detailed molecular mechanisms of interaction between different cells at different stages of the regeneration process, are poorly investigated and remain largely controversial. Despite the fact that immunomodulatory properties of MSCs are well documented in vitro, the molecular mechanisms that ensure the polarization of MSCs, i.e., switching the expression of proinflammatory cytokines to anti-inflammatory cytokines in vivo, are practically undefined.

Previously, we proposed to use the human umbilical cord as a source of MSCs (UCMSCs) to develop a potential therapeutic agent for the treatment of chronic diseases. MSCs obtained from the human umbilical cord after a normal 38-40-week gestation delivery (UCMSCs) were phenotypically characterized by expression of human surface antigens CD73, CD90, and CD105 and their capacity to differentiate into chondro-, osteo- and adipogenic lineages [20, 21]. The investigation of NK cell-mediated cytotoxicity showed that UCMSCs were not lysed in mixed cultures with recipient immunocompetent cells in vitro. The relative content of T and B lymphocytes, NK cells, and CD14+ lymphocytes also did not change in the presence of UCMSCs in mixed cultures [22]. Acute toxicity analysis of the UCMSCs revealed no adverse effects in rats after subcutaneous injection. In an acute murine wound model, a single local administration of UCMSCs did not affect the healing rates compared with the control group but reduced scab formation [23]. In the present work, we investigated the ability of the UCMSCs to regulate chronic inflammation. The objective of our investigation was to assess the safety and efficacy of UCMSC therapy in chronic wound/ulcer healing via the qualitative assessment of granulation tissue, evaluating the reduction in wound/ulcer size in patients with long-term nonhealing wounds.

## 2. Materials and Methods

### 2.1. Participants and Study Design

The safety and efficacy of human UCMSCs in patients with long-term nonhealing wounds was investigated by an open, randomized, placebo-controlled pilot study according to the protocol approved by the Ethical Committee of Pirogov Russian National Research Medical University (RNRMU). The study was developed at the Department of Purulent Surgery of Municipal Clinical Hospital №15 in Moscow. The procedures were conducted in accordance with the Declaration of Helsinki. The patients signed voluntary informed consent forms to participate in the study. One hundred eight patients with chronic wounds of various etiologies were enrolled in the study and were randomly allocated using a computer-generated randomization sequence to the UCMSC therapy group (59 patients) or the comparison placebo group (49 patients) according to the eligibility criteria (Table 1).

The clinical examination of patients included anamnesis, interviews, analysis of complaints, anthropometric measurements, control of blood pressure and heart rate, laboratory screening of clinical and biochemical blood parameters, microbiological examination of the wound, microscopic examination of wound smears on the neutrophil, lymphocyte, macrophage, and fibroblast ratios for determining the wound healing stage, ultrasound dopplerography of lower limb arteries, and clinical assessment of the wound bed and periwound tissues. Before joining the study, patients of both groups received a standard treatment approved for chronic wounds (surgical debridement, hydrocolloid dressings, conservative pharmacotherapy, and adequate anesthesia) for four weeks. The patients of the cellular therapy group received an additional injection of UCMSCs. Patients of the placebo group received an equal volume of sterile normal saline. The dynamics of the healing process were evaluated by the qualitative assessment of the granulation tissue condition, wound area measurement, estimation of microvascular blood flow by Doppler ultrasound, and measurement of the transcutaneous partial pressure of oxygen. The numerical rating scale (NRS) was used for pain intensity assessment. The parameters were measured 2 and 4 weeks after the start of treatment while patients stayed in the hospital. The patients then underwent further observation during the following year to reveal any long-term complications or wound recurrence.

### 2.2. Preparation of UCMSCs

UCMSCs were isolated from the human umbilical cord obtained after normal deliveries of 38-40-week gestation with the informed consent of donors free of infectious agents (HIV-1, HIV-2, and hepatitis B). UCMSC cultures were obtained as previously described [24]. Briefly, the disintegrated umbilical cord tissues were treated with 200 U/ml collagenase type I and 40 U/ml dispase in PBS for 2 h at 37°C. An equal volume of StemPro™ MSC culture medium (Gibco, USA) was then added. The suspension was centrifuged for 5 min at 260 g. The pellet was resuspended in StemPro™ MSC supplemented with 100 U/ml penicillin and 100 U/ml streptomycin and 2 mM L-glutamine. The suspension was transferred to 60 mm Petri dishes and cultured at 37°C in a humidified atmosphere containing 5% CO_2_ until ~70-80% confluence. The UCMSCs were then detached with 0.25% trypsin-EDTA (Sigma-Aldrich, USA) and replated at a 1 : 3 dilution under the same culture conditions. UCMSCs were analyzed for the expression of human surface antigens CD73, CD90, and CD105 (BD Biosciences, USA) by flow cytometry using a FACSCanto II flow cytometer (BD Biosciences). Their multilineage differentiation capacity was confirmed as described in [21].

### 2.3. Treatment Procedure

During the procedure, the patient was in a position that provided free and convenient access to the wound defect. Before cellular therapy, the nonhealing ulcers were first debrided to remove any necrotic and infected tissues, and the wound and periwound areas were treated with chlorhexidine antiseptic solution, followed by washing with sterile normal saline and air drying. An aliquot of 2 × 10^7^ UCMSCs was suspended in sterile normal saline and was diluted with autologous sera to an appropriate volume based on 1 ml of suspension per 10 cm^2^ of wound area. Next, 0.1 ml of UCMSC suspension was injected subcutaneously and intramuscularly around the periphery and inside of the wound/ulcer every 8-10 mm. After the procedure, an atraumatic nonabsorbent hydrogel dressing was used to cover the wound/ulcer area for 2-3 days. The dressing was changed every 2-3 days. Wounds were photographed with digital cameras before the treatment and during each follow-up posttreatment visit.

### 2.4. Assessment of Microvascular Blood Flow Parameters

A one-channel laser Doppler monitoring instrument BLF-21 (Transonic System Inc., USA) with surface detector «R» was used to evaluate microvascular blood flow. The measurements were performed before and 2 and 4 weeks after the treatment procedure within 1 min after the stabilization of the blood flow perfusion indicators in the first interdigital space on the back surface of the foot, on the arch area of the foot plantar surface, and on the upper third of the shin. At the same time, the transcutaneous oxygen measurement in the perifocal zone and the wound defect zone was performed using a TSM-400 (Radiometer, Denmark).

### 2.5. Statistical Analysis

The Kolmogorov-Smirnov (K-S) test was used to determine normality. Data were analyzed for statistical significance by Student's *t*-test if the samples were normally distributed. The Mann-Whitney *U* test was used to compare differences between two independent groups when the dependent variables were not normally distributed. The chi-squared (*χ*^2^) test was used for qualitative data analysis. The median (*M*) and interquartile range (25%; 75%) or the average and standard deviation were calculated based on the conclusions about the normality of the sample. *p* < 0.05 was considered to be significant. Statistica 6.0 software was used.

## 3. Results

In the first phase of clinical trials, the safety of the UCMSCs was assessed in 30 healthy (according to WHO criteria) volunteers aged 30-60 years. A single intradermal injection was performed with 2 × 10^7^ UCMSCs in 5 ml of sterile normal saline. The safety of the procedure and the occurrence of complications were evaluated on the first day after the injection and 2, 4, and 12 weeks later. No local or systemic adverse reactions were observed in patients in any period of observation. The ability of UCMSCs to initiate tissue repair at the site of chronic inflammation was investigated in patients with long-term nonhealing wounds.

One hundred eight patients between 30 and 80 years of age (average: 59.99 ± 12.26 years; median: 62 years) were enrolled in the clinical study. Among the tested patients, 52 (48.15%) were male and 56 (51.85%) were female. Out of the 108 patients, 8 (7.4%) were under 40 years old, 42 (38.9%) were 41 to 60, and 58 (53.7%) were 61 to 80. Among the ulcer-treated patients, 39 (36.11%) had trophic ulcers, 31 (28.7%) had diabetic foot, 4 (3.7%) had pressure ulcers, and 32 (29.6%) had poorly granulating wounds caused by mixed pathology. The duration of the nonhealing ulcers persisting before treatment ranged from 4 weeks to 24 months, with a mean of 9 months. The distribution of patients in the placebo group and the UCMSC therapy group by sex, age, and ulcer duration is shown in Table 2.

The patients involved in the study were randomized into 2 groups. The cellular therapy group consisted of 59 patients whose standard treatment was supplemented with a single UCMCS suspension injection around the periphery and inside of the wound/ulcer. The UCMSC therapy was accomplished after debridement of the ulcers in a single sitting within 5 min. The total UCMSC dose administered to the patient was 2 × 10^7^ cells. The observation of patients within a day after UCMSC treatment did not reveal any adverse effects, including local injection swelling, fever, or hypertensive crisis. The placebo group included 49 patients who received an additional injection of normal saline under the same conditions. Microbiological examinations of wound discharge were carried out at the time of patient enrolment. The analysis of wound microbial isolates revealed a predominance of gram-positive bacteria. *Staphylococcus aureus*, *Pseudomonas aeruginosa*, *Escherichia coli*, *Enterobacter*, *Enterococcus*, *Proteus mirabilis*, *Acinetobacter*, and *Alcaligenes faecalis* were detected sporadically, often as a mixed infection. During the observation period, neither significant changes in the spectrum of microbial flora nor increased microbial contamination was revealed in either group.

**Figure 2:**
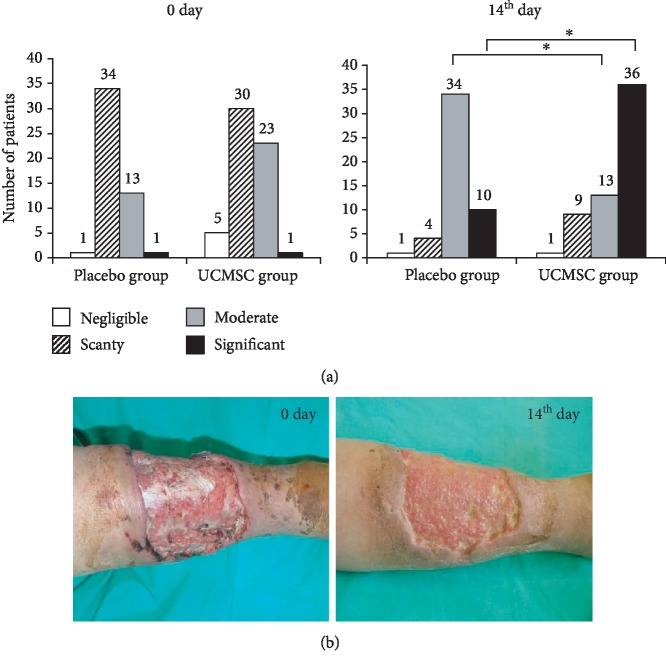
The dynamics of granulation tissue formation in chronic wounds. (a) The qualitative characteristics of granulation tissue in patients of the placebo and UCMSC groups before and 14 days after treatment (*χ*^2^ test; ^∗^reliability of the differences at *p* < 0.05). (b) representative images of wounds of the UCMSC group before and 14 days after cell therapy.

To assess the effectiveness of UCMSC therapy, we applied direct indicators of the dynamics of the wound healing process, including the qualitative characteristics of granulation tissue and dynamics of wound size reduction. The amount of red granulation tissue (percentage of the wound area) was assessed before the treatment and 14 days after by semiquantitative scoring (1—negligible 0-5%, 2—scanty 5-30%, 3—moderate 30-70%, and 4—profound 70-100%). The patients of both groups were divided into subgroups in accordance with these categories. The number of patients in subgroups of corresponding categories was compared using the *χ*^2^ test between the placebo and UCMSC groups before and after the treatment. Before the treatment, scanty or moderately developed granulation tissue was observed in both groups. The differences between the groups according to the *χ*^2^ test were statistically insignificant (*p* > 0.05). After treatment, the active growth of granulation tissue was observed in the UCMSC therapy group during the first week. After 15 days, patients with well-formed granulation tissue prevailed in this group. Meanwhile, the granulation tissue formation remained moderate in the placebo group. The differences between the groups according to the *χ*^2^ test were statistically significant (*p* < 0.01) (Figure 2(a)). Hence, the UCMSC injection stimulated the active growth and maturation of granulation tissue from the first day after treatment to create a favorable microenvironment for reepithelialization of the wound or for split-thickness skin grafting (Figure 2(b)).

**Figure 3:**
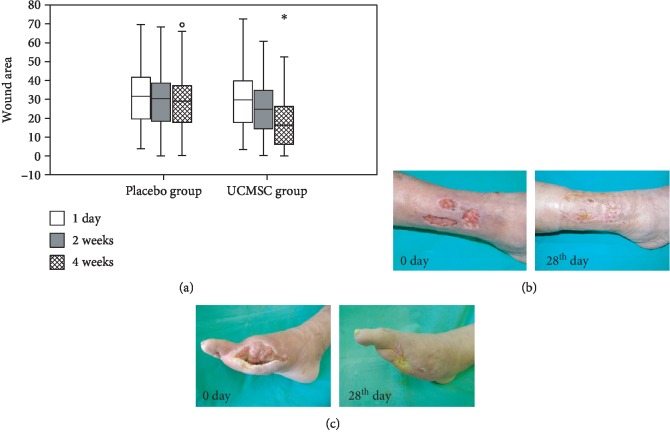
The dynamics of changes in wound sizes. (a) Wound area on the 1st, 14th, and 28th days of the study in the placebo and UCMSC groups. Data are presented as the median, 25% and 75% quartiles, and minimum and maximum (*U* test; ^∗^reliability of the differences at *p* < 0.05; “°” symbol indicates an outlier from the data set). (b) Reepithelialization of the wound in a patient from the UCMSC group 14 days after cell therapy. (c) Wound contraction in a patient from the UCMSC group 14 days after cell therapy.

Before treatment, the wound areas (length × width) of the placebo and UCMSC therapy groups were comparable, averaging 31.49 (interquartile interval—19.35/41.54) cm^2^ and 29.07 (interquartile interval—18.17/39.9) cm^2^, respectively. The differences between groups were statistically insignificant (*U* test, *p* > 0.05). After the UCMSC treatment, a significant improvement in wound healing resulted in a reduction of wound size, which was more clearly observed in the cellular therapy group in comparison with the placebo group. Four weeks after treatment, the wound area in the UCMSC group averaged 15.75 (interquartile interval—5.33/26.19) cm^2^, while the wound area was 27.60 (interquartile interval—17.6/35.84) cm^2^ for the placebo group, and the differences between the groups were statistically significant (*U* test, *p* < 0.01) (Figure 3(a)). The wound healing rate in the two groups also differed significantly. From 1 to 14 days, the wound healing rate was 2.19 ± 1.58% per day in the UCMSC group and 0.73 ± 1.2% per day in the placebo group (*U* test, *p* < 0.01). The dynamics of the wound area regression was the most pronounced due to edge epithelialization (Figure 3(b)) and contraction (Figure 3(c)).

**Figure 4:**
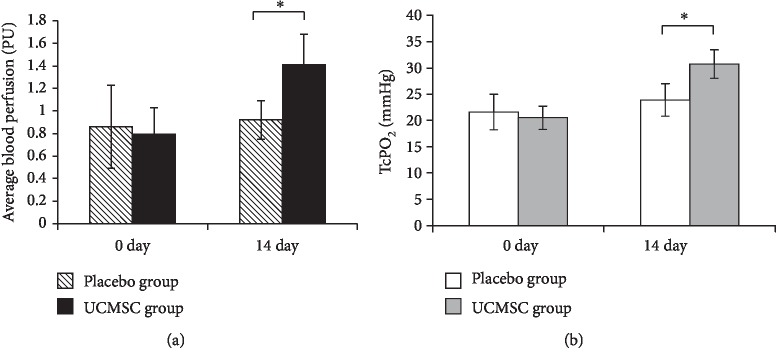
The quantitative analysis of microvascular hemodynamic parameters of periwound tissues. (a) Transcutaneous oxygen pressure (TcPO_2_) in patients of the placebo and UCMSC groups before and 14 days after treatment. (b) Average blood perfusion in patients of the placebo and UCMSC groups before and 14 days after treatment (*t*-test; ^∗^reliability of the differences at *p* < 0.05).

The functional status of periwound tissue was estimated by quantification analysis of laser Doppler data of microvascular hemodynamics, including average blood perfusion, amplitude, frequency, relative velocity of blood flow, and oxygen saturation. Our finding showed that 2 weeks after treatment, the indicators of microcirculation significantly improved in the UCMSC group. The pretreatment average blood perfusion was 0.86 ± 0.37 perfusion units (PU) and 0.79 ± 0.24 PU in the placebo and UCMSC groups, respectively. Differences between the groups were statistically insignificant (*t*-test, *p* = 0.367). Two weeks after treatment, the average blood perfusion changed slightly to 0.92 ± 0.17 in the placebo group, while it was significantly increased to 1.41 ± 0.27 PU in the UCMSC group (*t*-test, *p* < 0.05) (Figure 4(a)). Transcutaneous oxygen pressure (TcPO_2_) measurements showed the same dynamics. At two weeks after treatment, the level of oxygen pressure in the perifocal zone and the wound defect zone changed slightly, from 21.6 ± 3.4 mmHg to 23.9 ± 3.1 mmHg, in the placebo group. In the UCMSC group, a significant increase of TcPO_2_ values was detected compared with the initial observations, from 20.5 ± 2.2 mmHg to 30.7 ± 2.7 mmHg (Figure 4(b)). No statistically significant differences were found in the amplitude, frequency, or relative velocity in microvascular hemodynamics of the groups. Four weeks after treatment, microvascular hemodynamic parameter values changed slightly compared with the previous measurement (data not shown).

During the observation period, the reduction in pain was noted, which was more pronounced in the UCMSC group, probably due to the anti-inflammatory property of the cells. Four weeks after treatment, a significant improvement (complete wound closure or reduction in wound size for more than 90% from the initial value) was achieved in 17 cases (15.7% of total enrolled patients), including 13 cases (22%) in the UCMSC group and 4 cases (8.2%) in the placebo group. Moderate improvement (reduction in wound size less than 90% but more than 25% from the initial value) was found in 70 cases (64.8% of the total enrolled patients), including 41 cases (69.5%) in the UCMSC group and 29 cases (59.2%) in the placebo group. Negative outcomes (increase in wound size compared with the initial value) or insignificant improvement (reduction in wound size less than 25% from the initial value) was observed in 21 cases (19.4% of the total enrolled patients), including 5 (8.5%) cases in the UCMSC group and 16 cases (32.6%) in the placebo group (Table 3).

Further observations within the 1-year period revealed no prominent adverse events nor any long-term complications in patients from the UCMSC group.

## 4. Discussion

The long-term nonhealing or chronic wounds caused by neuropathy, ischemia, trauma, or mixed pathology are often difficult to treat. The main goal of such treatments is to obtain wound closure. However, despite the use of various therapies, chronic wounds often take a long time to heal. MSCs have gained considerable attention as promising therapeutic agents for treating chronic wounds. Previous studies and clinical trials have demonstrated that MSCs from bone marrow and adipose tissue are capable of promoting cutaneous wound healing in animals [25–27] and patients with chronic wounds [28, 29]. MSCs isolated from the umbilical cord (Wharton's jelly) may also become a perspective therapeutic agent for long-term nonhealing wounds. Here, we used the human umbilical cord after normal delivery at 38-40 weeks of gestation from healthy donors. The umbilical cord is an ethical and noncontroversial source of MSCs. The umbilical cord collection procedure is noninvasive and painless. UCMSCs have many advantages, including improved proliferative capacity, greater multipotentiality, and retardation of senescence compared with adult MSCs [30, 31]. The laboratory comparison of UCMSCSs with MSCs isolated from bone marrow according to morphology, expression of surface markers CD105, CD73, and CD90, and ability to differentiate into adipocytes, chondroblasts, and osteoblasts demonstrated their similarity in accordance with the minimal criteria proposed for MSCs by ISCT [20, 21, 32]. Preliminary animal studies have suggested that the infusion of MSCs from bone marrow and adipose tissue promotes healing of full-thickness wounds in mice [26, 33], rats [34], rabbits [27], sheep [35]. Enhanced wound healing in diabetic mice was also observed after subcutaneous administration of human UCMSCs. But the healing was even better and more rapid after administration of conditioned media from UCMSCs compared to that achieved by UCMSC transplantation [36]. Our investigation of acute wound healing in mice demonstrated that the administration of UCMSCs did not accelerate wound closure compared with the control group. This animal model has some limitations, because wound healing in mice occurs quite rapidly. However, we revealed that UCMSCs have major effects on granulation tissue formation. UCMSCs induced the formation of red and moist granulation tissue in mice, while the wounds were healed under the scab in the control group. We also noticed that UCMSCs promoted hair follicle neogenesis in large full-thickness wounds in mice. Hence, these results demonstrated that UCMSCs have the capacity to promote some tissue repair processes [23]. The present study was initiated to assess the safety and efficacy of UCMSC therapy in patients with long-term nonhealing wounds.

A recent meta-analysis of 36 prospective clinical trials summarizing the safety of MSC administration was unable to detect associations between MSC treatment and development of acute infusional toxicity, organ system complications, infection, death, or malignancy [37]. Our research has also found no adverse consequences associated with a single intradermal infusion of UCMSCs in healthy patients. Hence, UCMSC therapy could be qualified as a safe treatment procedure. Previous clinical studies examining the effectiveness of MSC therapy in patients with various acute and chronic skin injuries such as diabetic skin ulcers, radiation, and thermal burns have shown the improvement of the wounds within days following administration of MSCs, characterized by a decrease in wound size, increase in the vascularity of the dermis, and increased dermal thickness of the wound bed [28, 29, 38, 39]. The type of MSCs used for chronic wound therapy in these studies varies widely: cultured MSCs from bone marrow and adipose tissue, bone marrow mononuclear cells, and stromal vascular fraction derived from adipose tissue. The number of cells administered, routes of administration, evaluation of wound healing, and follow-up times are also substantially different across studies. Currently, there are no published results of clinical studies utilizing UCMSCs in wound healing. Our research demonstrated that UCMSC treatment in patients with long-term nonhealing wounds induces active growth of granulation tissue and increases blood circulation and oxygen saturation of periwound tissues, thereby contributing to the reepithelialization and acceleration of wound healing compared with the placebo group. The healing outcomes were better in patients with diabetic foot and ulcers caused by multilevel occlusions of small-caliber arteries which could not be removed surgically. Analysis of long-term outcomes (one-year follow-up) in patients undergoing UCMSC therapy showed that in most cases, the UCMSC treatment resulted in complete wound healing or a significant reduction in the area of the wound without any recurrence. The success of the UCMSC treatment considerably depended on the size of the wound/ulcer, the stage, the degree of hemodynamic disorders in arteries of the lower limbs, and the level of bacterial infection.

A variety of mechanisms have been proposed to explain the beneficial effect of UCMSC treatment for long-term nonhealing wounds, including neovascularization, cell differentiation, and immunomodulation. Neovascularization is necessary for growing and sustaining granulation tissue, the formation of which is one of the most important processes in wound healing. Previous research has suggested that UCMSCs can enhance angiogenesis by promoting the formation of capillary-like structures in vitro or by increasing capillary density in vivo through the secretion of VEGF, KGF, PDGF, and chemokines [36, 40]. The trophic effects are implemented by the secretion of growth factors by UCMSCs, including PDGF, TGF-*β*, and bFGF [41–43]. The UCMSC-mediated secretion of matrix proteins (such as fibronectin and collagen) creates a microenvironment favorable for epithelial growth [18, 19, 44]. While the ability of UCMSCs to differentiate into osteoblasts, chondrocytes, myocytes, and adipocytes is well described in vitro [21, 45, 46], their multipotent properties have not been clearly demonstrated in vivo. Moreover, the physiological relevance of their differentiation potential into additional lineages, including cardiomyocytes, endothelial cells, hepatocytes, neural cells, keratinocyte, fibrocytes, endothelial cells, and pericytes, is still to be determined. However, this obvious mechanism of accelerating the wound healing process cannot be ignored. The effectiveness of UCMSC therapy can also be explained by their immunomodulatory properties. In vitro experiments have shown that UCMSCs, as well as MSCs isolated from bone marrow and adipose tissue, possess the ability to reduce the expression of proinflammatory cytokines by immune cells [47–49] and to inhibit the proliferation of activated T lymphocytes [50–52]. They also suppress the differentiation and maturation of dendritic cells [53, 54] and mediate the induction of a regulatory phenotype in activated T lymphocytes (Treg) [55, 56]. Perhaps the immunomodulatory function of UCMSCs can change in vivo when a patient's MSCs migrate to the site of inflammation from adjacent or distant tissues and/or proliferate there and when allogeneic MSCs cultured ex vivo are injected into the site of inflammation. Moreover, autologous MSC potency may be blunted in patients with chronic systemic illness, and administration of exogenous allogenic UCMSCs can improve endothelial function and vascular reactivity through stimulation of endogenous endothelial progenitor cell mobilization [40, 57] and induction of the appearance of alternatively activated anti-inflammatory macrophages. This assumption is supported by the following considerations. It is known that the differences in cellular composition and cytokine microenvironments are significant in acute and chronic inflammation.

The main cells of acute inflammation are neutrophils, which clean the wound of pathogens and the debris from the extracellular matrix. Chemokines produced by neutrophils attract monocytes to the site of inflammation and induce their differentiation into macrophages. Macrophages are the key cells that determine the effectiveness of the inflammatory process and the transition to subsequent phases of regeneration. In the early phase of inflammation, macrophages secrete proinflammatory mediators such as IL-1*β*, IL-6, IL-12, IL-18, and TNF-*α*. This functional phenotype of proinflammatory macrophages is called M1. In later stages of repair, macrophages acquire the anti-inflammatory phenotype M2 and secrete the anti-inflammatory cytokines IL-10, CCL17, CCL18, CCL22, CCL24, EGF, TGF-*β*, and IGF1 [58]. This process leads to normal wound healing. The influence of some factors can lead to defects in the phenotypic switch in macrophages at the site of inflammation, whereby the tissue infiltrate consists of proinflammatory macrophages in M1. The resolution of acute inflammation is delayed, and chronic inflammation develops [59]. In this case, the elimination of factors affecting wound healing and/or the administration of substances capable of stimulating the switch of M1 macrophages toward M2 phenotype are required for triggering the transition from delayed acute inflammation to the subsequent phases of tissue regeneration. IDO and IL-6 secreted by MSCs cause an increase in the expression of IL-4 R, which contributes to the polarization of macrophages toward the phenotype M2 [60–63]. Recent studies in vitro have shown that MSCs are able to induce the appearance of alternatively activated macrophages that are different from macrophages with M1 or M2 phenotype. Such alternatively activated macrophages are characterized by a high level of expression of the CD206 and CD163 phagocytic receptors [64], as well as secretions of IL-10 and TGF-*β*. Such macrophages suppress the expression of natural killer (NK) cell activation markers (NKp44, CD69, and CD25) and IFN-*γ* secretion. This is in contrast to M2 macrophages, which suppress only IFN-*γ* secretion. Such alternatively activated macrophages can inhibit the proliferation of CD8+ T lymphocytes and promote the induction of CD25highFoxp3+ Treg cells [65]. Thus, external administration of UCMSCs may induce the appearance of M2 macrophages at the site of chronic inflammation and trigger the process of normal wound healing in vivo. This has been confirmed by our research.

Taken together, the findings from preclinical and clinical studies have demonstrated that UCMSCs are an effective and safe method for treating long-term nonhealing wounds. Locally delivered allogeneic UCMSCs can contribute to wound repair and may be a resource for regenerative medicine.

## Figures and Tables

**Table 1:** Enrollment criteria.

Inclusion criteria	Exclusion criteria
Presence of chronic wounds at least 4-week duration	Pregnancy and lactation period
	Concomitant malignancy
Chronic wound arrested in an inflammatory stage	Concomitant chronic diseases in the decompensation stage
18 to 90 years old	Patients in terminal state
Voluntary informed consent	2-year alcoholism or drug addiction prior to the enrollment in the study
	Sepsis, septicopyemia
	Persistence anaerobic infection in the wound

**Table 2:** The composition of patients by age, sex, and duration of ulcer disease in both groups.

		Placebo group	UCMSC group	Total
Age (*t*-test, *p* = 0.211)	Mean ± std	61.85 ± 11.84	58.5 ± 12.33	59.99 ± 12.26
	*M*	64	60	62
	25%/75% q	54/72	49/68	52/70

Sex (*t*-test, *p* = 0.701)	Male; *n* (%)	24 (49%)	28 (47.5%)	52 (48.1%)
	Female; *n* (%)	25 (51%)	31 (52.5%)	56 (51.9%)

Duration of ulcers, months (*U* test, *p* = 0.745)	Mean ± std	7.87 ± 5.9	8.66 ± 6.88	8.30 ± 6.16
	*M*	6	6	6
	25%/75% q	3/12	2.5/12	3/12

Mean ± std: average and standard deviation; *M*: median; 25%/75% q: interquartile range (25%; 75%).

**Table 3:** Cases of wound improvement and closure among the patients 4 weeks after the treatment (*χ*^2^ test, *p* < 0.05).

Wound improvement		Placebo group	UCMSC group	Total
Complete wound closure or significant improvement	Patients in group	4	13	17
	% in group	8.2%	22%	15.7%
	% in subcategory	23.5%	76.5%	100%

Moderate improvement	Patients in group	29	41	70
	% in group	59.2%	69.5%	64.8%
	% in subcategory	41.4%	56.6%	100%

Negative outcomes or insignificant improvement	Patients in group	16	5	21
	% in group	32.6%	8.5%	19.5%
	% in subcategory	76.2%	23.8%	100%

## Data Availability

The primary data used to support the findings of this study are restricted by the Ethical Committee of Pirogov Russian National Research Medical University in order to protect patient privacy. Data are available from Professor Victor. A. Stupin (RNRMU, Ostrovityanova str., 1, Moscow 117997, Russia, stvictor@bk.ru) for researchers who meet the criteria for access to confidential data.
